# Evaluating the impact of 18F-FDG-PET-CT on risk stratification and treatment adaptation for patients with muscle-invasive bladder cancer (EFFORT-MIBC): a phase II prospective trial

**DOI:** 10.1186/s12885-021-08861-x

**Published:** 2021-10-18

**Authors:** Flor Verghote, Lindsay Poppe, Sofie Verbeke, Piet Dirix, Maarten Albersen, Gert De Meerleer, Charlien Berghen, Piet Ost, Geert Villeirs, Pieter De Visschere, Kathia De Man, Daan De Maeseneer, Sylvie Rottey, Charles Van Praet, Karel Decaestecker, Valérie Fonteyne

**Affiliations:** 1 Department of Radiotherapy-Oncology, Ghent University Hospital, Ghent, Belgium; 2 Department of Human structure and Repair, Ghent University, Ghent, Belgium; 3 Department of Pathology, Ghent University Hospital, Ghent, Belgium; 4 Department of Radiation-Oncology, Iridium Network, Antwerp, Belgium; 5 Department of Urology, University Hospitals Leuven, Leuven, Belgium; 6 Department of Radiotherapy-Oncology, University Hospitals Leuven, Leuven, Belgium; 7 Department of Radiology, Ghent University Hospital, Ghent, Belgium; 8 Department of Nuclear Medicine, Ghent University Hospital, Ghent, Belgium; 9 Department of Medical Oncology, Ghent University Hospital, Ghent, Belgium; 10 Department of Urology, Ghent University Hospital, Ghent, Belgium

**Keywords:** Muscle-invasive bladder cancer, Primary staging, ^18^F-FDG-PET-CT, Distant metastasis, Oligometastasis, Neo-adjuvant chemotherapy, Metastasis-directed therapy, Stereotactic body radiation therapy, Immunotherapy, Overall survival

## Abstract

**Background:**

The outcome of patients with muscle-invasive bladder cancer (MIBC) remains poor, despite aggressive treatments. Inadequate primary staging, classically performed by computed tomography (CT)-imaging, could lead to inappropriate treatment and might contribute to these poor results. Although not (yet) adapted by international guidelines, several reports have indicated the superiority of ^18^F-fluorodeoxyglucose-positron emission tomography-CT (^18^F-FDG-PET-CT) compared to CT in the detection of lymph node and distant metastases. Thereby the presence of extra-vesical disease on ^18^F-FDG-PET-CT has been correlated with a worse overall survival. This supports the hypothesis that ^18^F-FDG-PET-CT is useful in stratifying MIBC patients and that adapting the treatment plan accordingly might result in improved outcome.

**Methods:**

EFFORT-MIBC is a multicentric prospective phase II trial aiming to include 156 patients. Eligible patients are patients with histopathology-proven MIBC or ≥ T3 on conventional imaging treated with MIBC radical treatment, without extra-pelvic metastases on conventional imaging (thoracic CT and abdominopelvic CT/ magnetic resonance imaging (MRI)). All patients will undergo radical local therapy and if eligible neo-adjuvant chemotherapy. An ^18^F-FDG-PET-CT will be performed in addition to and at the timing of the conventional imaging. In case of presence of extra-pelvic metastasis on ^18^F-FDG-PET-CT, appropriate intensification of treatment with metastasis-directed therapy (MDT) (in case of ≤3 metastases) or systemic immunotherapy (> 3 metastases) will be provided. The primary outcome is the 2-year overall survival rate. Secondary endpoints are progression-free survival, distant metastasis-free survival, disease-specific survival and quality of life. Furthermore, the added diagnostic value of ^18^F-FDG-PET-CT compared to conventional imaging will be evaluated and biomarkers in tumor specimen, urine and blood will be correlated with primary and secondary endpoints.

**Discussion:**

This is a prospective phase II trial evaluating the impact of ^18^F-FDG-PET-CT in stratifying patients with primary MIBC and tailoring the treatment accordingly. We hypothesize that the information on the pelvic nodes can be used to guide local treatment and that the presence of extra-pelvic metastases enables MDT or necessitates the early initiation of immunotherapy leading to an improved outcome.

**Trial registration:**

The Ethics Committee of the Ghent University Hospital (BC-07456) approved this study on 11/5/2020. The trial was registered on ClinicalTrials.gov (NCT04724928) on 21/1/2021.

**Supplementary Information:**

The online version contains supplementary material available at 10.1186/s12885-021-08861-x.

## Background

Bladder cancer is the 10th most frequently diagnosed cancer and ranks 14th in causes of cancer-related death worldwide [1]. As the incidence of bladder cancer increases steadily with age and life expectancy improves, the number of bladder cancer patients of whom approximately 30% are diagnosed with muscle-invasive bladder cancer (MIBC) [2], is expected to increase in the future [3]. Despite an aggressive treatment with neo-adjuvant chemotherapy [4] followed by either radical cystectomy (RC) or trimodality therapy (TMT) [5], the outcome of MIBC patients remains poor with 2- to 5-year overall survival (OS) rates of ±60 and 50%, respectively [3, 4] and a 2-year disease free survival rate of ±64% [3]. Inadequate primary staging probably contributes to these poor results as adequate staging and appropriate treatment are closely linked.

Staging is classically done by computed tomography (CT) of the chest, abdomen and pelvis [6]. Although several reports indicate the superiority of ^18^F-fluorodeoxyglucose-positron emission tomography-CT (^18^F-FDG-PET-CT) compared to CT in the detection of lymph node as well as distant metastases [7], ^18^F-FDG-PET-CT is currently not recommended in international guidelines [6].

The presence of metastases on ^18^F-FDG-PET-CT has been correlated with survival rates. When compared to patients without metastatic lesions on ^18^F-FDG-PET-CT, MIBC patients with metastases on ^18^F-FDG-PET-CT had inferior OS and disease-specific survival (DSS). At 2 years the difference increased to 35% for both OS and DSS in favor of ^18^F-FDG-PET-CT-negative MIBC patients [8]. This suggests that there is a role for ^18^F-FDG-PET-CT in risk stratification to guide optimal treatment strategy.

For patients with metastatic disease on ^18^F-FDG-PET-CT, we hypothesize that treatment intensification improves outcome. In analogy with other tumor types [9–11], trials -although limited in patient number- have demonstrated that metastasis-directed therapy (MDT) of a limited number of metastases results in improved and durable disease control [12]. Similarly, patients with multiple metastases on ^18^F-FDG-PET-CT can benefit from earlier initiation of systemic immunotherapy [13].

To our knowledge, there is currently no prospective trial evaluating the impact of ^18^F-FDG-PET-CT implementation in the staging of patients with MIBC, to stratify patients and guide further treatment decisions in order to improve the outcome of patients with primary MIBC.

## Methods/design

This study is approved by the Ethics Committee of the Ghent University Hospital (BC-07456) and the Belgian Federal Agency for Nuclear Control (PK-0061377). The trial is registered on ClinicalTrials.gov (NCT04724928).

**Fig. 1:**
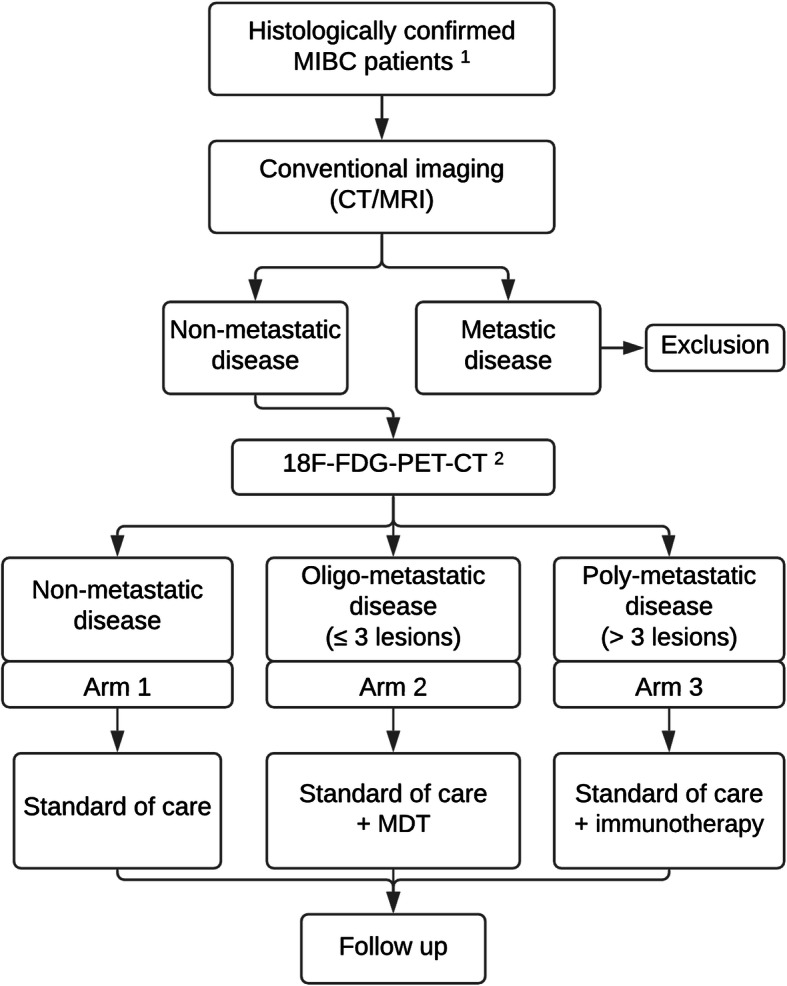
Overview of the EFFORT-MIBC study design. (1) Inclusion and exclusion criteria need to be fulfilled to be included in the study. (2) Stratification into treatment arms is based on the ^18^F-FDG-PET-CT result. In case of neo-adjuvant chemotherapy, stratification is based on the results of both ^18^F-FDG-PET-CT’s (e.g. prior to and after neo-adjuvant chemotherapy). Abbreviations: MIBC: muscle-invasive bladder cancer; CT: computed tomography; MRI: magnetic resonance imaging; ^18^F-FDG-PET-CT: ^18^F-fluorodeoxyglucose-positron emission tomography-computed tomography; MDT: metastasis-directed therapy

In this multicentric prospective phase II trial, patients with MIBC will be offered ^18^F-FDG-PET-CT in addition to and at the timing of the conventional imaging (thoracic CT and abdominopelvic CT/ magnetic resonance imaging (MRI)), to guide further treatment after radical local therapy. In case of presence of metastasis on ^18^F-FDG-PET-CT, appropriate intensification of treatment with MDT or immunotherapy will be provided. A flowchart presenting the different steps from inclusion until follow-up (as described below) is presented in Fig. 1. Items from the World Health Organization Trial Registration Data Set are addressed in an additional file (see additional file 1).

### Objectives

The primary endpoint is the 2-year overall survival (OS) rate defined as the percentage of patients alive at 2 years after diagnosis of MIBC. OS is calculated from time of diagnosis until death due to MIBC or other causes.

Secondary endpoints are progression-free survival (PFS, defined as time of diagnosis until progression: i.e. local (T2-T4 in case of of TMT)/locoregional recurrence or extra-pelvic metastases), distant metastasis-free survival (DMFS, defined as time of diagnosis until occurrence of distant metastasis on repeated imaging), disease-specific survival (DSS, defined as time of diagnosis until death due to MIBC) and quality of life (QOL) evaluated by the general EORTC QOL questionnaire QLQ-C30 (version 3) and the bladder cancer specific module QLQ-BLM30. The added diagnostic value of ^18^F-FDG-PET-CT compared to conventional imaging will be evaluated. If neo-adjuvant chemotherapy is administered, the treatment response will be evaluated on the repeated ^18^F-FDG-PET-CT. A biopsy specimen of the bladder, obtained after transurethral resection of the bladder (TURb) as well as urine and blood samples will be collected for validation of predictive biomarkers by evaluating the correlation between response to therapy and outcome (PFS, DMFS, DSS and OS) with in literature reported biomarkers determined on biopsy specimen of the bladder, obtained after TURb.

### Inclusion criteria

- Histopathology-proven MIBC on TURb or ≥ T3 on conventional imaging treated with MIBC radical treatment
- T1–4 N0–3 M0 MIBC on conventional imaging (thoracic CT and abdominopelvic CT/ MRI)
- Age > 18 years
- WHO 0–2
- Willingness to undergo ^18^F-FDG-PET-CT
- Willingness to undergo MDT or immunotherapy, in case of diagnosis of oligometastatic or polymetastatic disease on ^18^F-FDG-PET-CT, respectively
- Willingness and ability to provide a signed informed consent according to ICH/GCP and national/local regulations

### Exclusion criteria

- Presence of distant metastasis on conventional imaging (thoracic CT and abdominopelvic CT/ MRI)
- Refusal of or having contraindications to ^18^F-FDG-PET-CT
- Refusal of MDT or immunotherapy
- Prior radiotherapy unabling MDT
- Contraindications to radiotherapy (including active inflammatory bowel disease)
- Contraindications to immunotherapy
- Other primary tumor diagnosed < 5 years ago and for which treatment is still required, except for diagnosis of non-metastatic prostate cancer at time of diagnosis of MIBC or non-melanoma skin cancer.

### Evaluation and inclusion

Patients who were recently diagnosed with MIBC on TURb and who are considered for curative treatment, will be informed of this clinical study if eligible at the departments of urology, radiation oncology, medical oncology or at the multidisciplinary consultations as carried out in some participating centers. The decision to participate will be entirely voluntary. Eligible patients who decide not to participate will be offered standard of care treatment.

### Intervention

#### Imaging

Conventional imaging, consisting of standard thoracic CT and abdominopelvic CT (or MRI), will be performed prior to the radical local therapy. In case of neo-adjuvant chemotherapy, conventional imaging will be performed prior to and after neo-adjuvant chemotherapy. After the second cycle of chemotherapy, MRI bladder will be performed to evaluate provisional therapy response. In addition to and at timing of the conventional imaging patients will receive an ^18^F-FDG-PET-CT. We aim to perform the ^18^F-FDG-PET-CT within 1 month of the thoracic CT and abdominopelvic CT (or MRI).

The ^18^F-FDG-PET-CT procedure is described in detail in an additional file (see Additional file 2).

Conventional imaging images will be evaluated by experienced radiologists in uro-oncology. ^18^F-FDG-PET-CT images will be reviewed and analyzed by a nuclear medicine physician and a radiologist experienced in reading PET-CT images. Evaluation of the ^18^F-FDG-PET-CT images will occur blinded to the conventional imaging or vice versa in case the ^18^F-FDG-PET-CT is performed first.

After ruling out presence of distant metastases on conventional imaging and evaluation of the ^18^F-FDG-PET-CT’s, patients will be allocated to the appropriate treatment arm:

- Arm 1: ^18^F-FDG-PET-CT ➜ T1–4 N0–3 M0
- Arm 2: ^18^F-FDG-PET-CT ➜ T1–4 N0–3 M1 (≤3 metastases)
- Arm 3: ^18^F-FDG-PET-CT ➜ T1–4 N0–3 M1 (> 3 metastases)

If no neo-adjuvant chemotherapy is administered, group allocation is based on the findings of the single ^18^F-FDG-PET-CT. In case of neo-adjuvant chemotherapy, group allocation is based on ^18^F-FDG-PET-CT with the highest number of distant metastases. Thus, patients who show a decrease of the total number of metastases or a complete response on the second ^18^F-FDG-PET-CT, will be allocated based on the result of their first ^18^F-FDG-PET-CT.

#### Treatment

All included patients will receive a standard of care approach which involves either RC with extended pelvic lymph node dissection (ePLND) or TMT consisting of a visible complete TURb and radiochemotherapy. Radiochemotherapy consists of moderately hypofractionated radiotherapy to a dose of 55 Gy in 20 fractions to the bladder, in combination with a radiosensitizer. In case of clinically node-positive disease, the pelvic nodal areas are included in the radiotherapy field. Decision of local therapy is at the discretion of the patient unless there is a contraindication for one treatment option. If the patient is eligible, neo-adjuvant chemotherapy will be administered.

^18^F-FDG-PET-CT information concerning the pelvic lymph nodes will be used to guide local treatment i.e. adapting the ePLND template or radiotherapy field, if feasible.

According to the risk-group allocation, treatment will be intensified by adding MDT or immunotherapy, in treatment arm 2 or 3, respectively. Patients will not be randomized. Both surgery and stereotactic body radiation therapy (SBRT) can be applied as MDT for oligometastases. The SBRT procedure is described in detail in an additional file (see Additional file 3). In treatment arm 3, systemic immunotherapy will be initiated in case of ^18^F-FDG-PET-CT detected polymetastatic disease. The systemic immunotherapy protocol can be adjusted following changes in guidelines and/or reimbursement criteria.

In case of diagnosis of metastases on the conventional imaging before local treatment is started, the patient is excluded from the trial and metastatic bladder cancer standard of care is initiated.

### Follow up and data collection

**Fig. 2:**
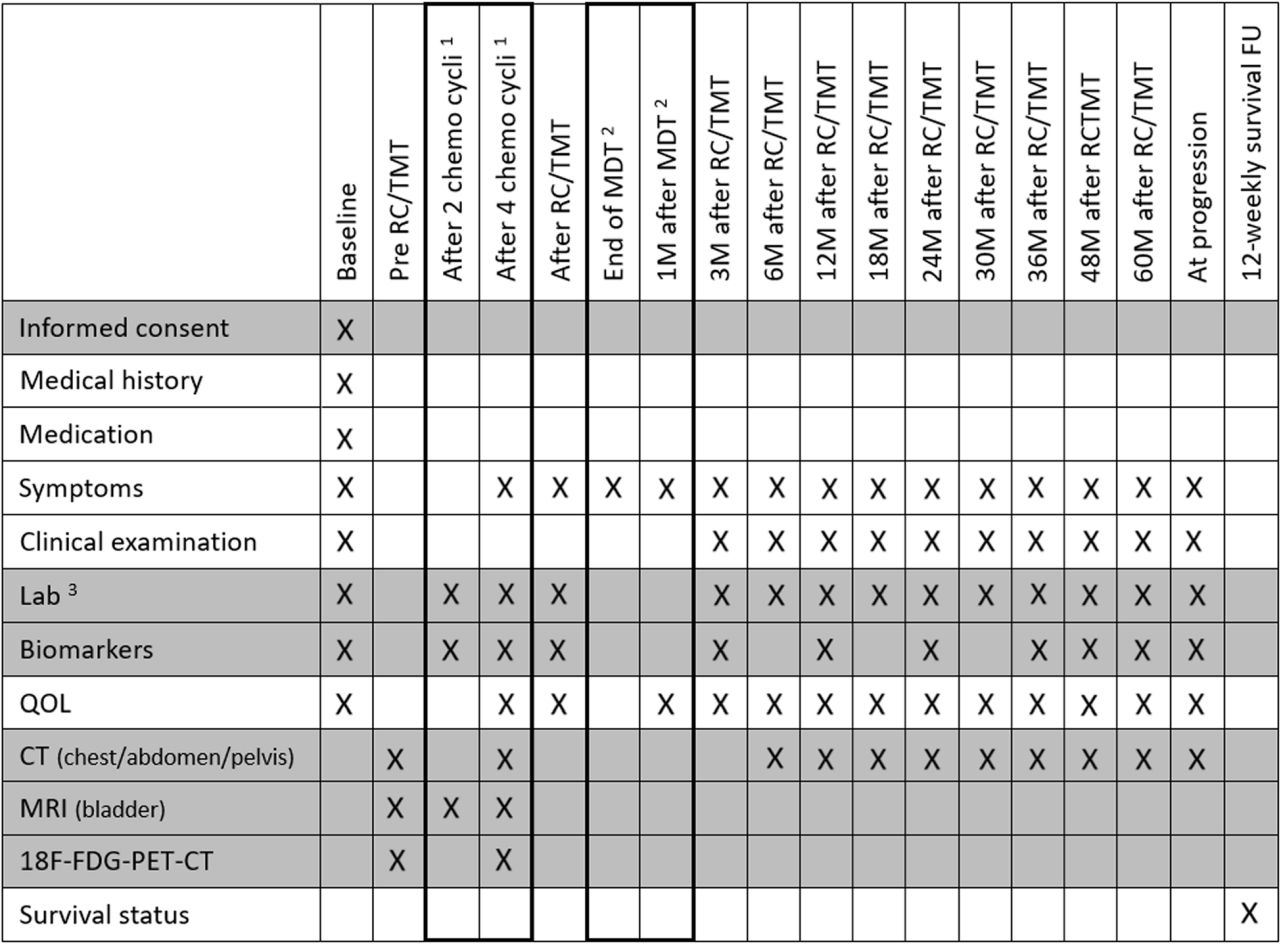
Schedule of the follow-up moments and associated assessments and investigations. (1) Follow-up specific for patients receiving neo-adjuvant chemotherapy. (2) Follow-up specific for patients in treatment arm 2. (3) At predefined follow-up visits a standard blood control (including erythrocytes, leucocytes (including formula), thrombocytes, sedimentation, creatinine, electrolytes, liver/renal and inflammatory parameters) is performed. Abbreviations: QOL: quality of life; CT: computed tomography; MRI: magnetic resonance imaging; ^18^F-FDG-PET-CT: ^18^F-fluorodeoxyglucose-positron emission tomography-computed tomography; RC: radical cystectomy; TMT: trimodality treatment; MDT: metastasis-directed therapy; M: month(s); FU: follow-up

An overview of the different follow-up moments and associated assessments and investigations is presented in Fig. 2. Adverse events (AE) will be assessed using the Common Terminology Criteria for Adverse Events version (CTCAE) 5.0 [14].. Patients will be instructed by the investigator to report the occurrence of any AE. The investigator assesses and records all AE observed during the AE reporting period from inclusion until 5 years after inclusion. QOL will be assessed using the European Organisation for Research and Treatment of Cancer (EORTC) QLQ-C30 [15] and QLQ-BLM30 [16] questionnaires. Imaging studies are done conform standard of care. In case of TMT a routine cystoscopy is advocated every 3 months during the first year of follow-up and 6-monthly thereafter until 5 years. Patients will be followed up until death or disease progression defined as per: Response Evaluation Criteria in Solid Tumors (RECIST)-criteria [17]. Once disease progression has been confirmed, survival status will be assessed 3-monthly until death, withdrawal of consent or the end of the study, whichever occurs first. Patients who are no longer followed in the center of inclusion or who refuse follow-up visits but are willing to have telephone follow-up will be contacted by phone at the scheduled follow-up time points.

### Data management and confidentiality

All study data will be handled in accordance with the law on General Data Protection Regulation (GDPR) and institutional rules. The collection and processing of personal data from subjects enrolled in this study will be limited to those data that are necessary to fulfill the objectives of the study. Study-related data of the patient will be provided in a coded manner to Ghent University Hospital. A sequential unique and coded study ID number will be attributed to each patient included into the trial, to maintain participants confidentiality. Identification of patients must be guaranteed at the center of inclusion. In order to avoid identification errors, the year of birth and the unique Study ID Number need to be provided on the case report form. All data will be collected and managed using REDCap (Research Electronic Data Capture) electronic data capture tools hosted at Ghent University Hospital, a secure web-based application designed to support data capture for research studies [18]. Patient data will be stored for 25 years.

### Statistical analyse

#### Sample size

This is a multicentric prospective phase II study in which 156 patients will be enrolled. A confidence interval for the 2-year OS was estimated using the Kaplan Meier survival estimates and corresponding confidence intervals (type ‘log-log’). These Kaplan Meier survival estimates are based on binomial proportions. When the true proportion of survival is 63% for arm 2 + 3, a sample size of 35 patients (39 when taking into account 10% drop-out rate) in group 2 + 3 yields 80% power to show that the proportions of patients surviving at 2 years, is 38% or more using an exact binomial test at alpha level 5% [12]. We expect 1/4 patients to have a positive ^18^F-FDG-PET-CT, based on the unpublished observations of Fonteyne et al.

#### Subsequent analyses

Descriptive statistics will be used to summarize patient characteristics and toxicity per treatment group. Progression and survival are defined as mentioned above and calculated from time of diagnosis to disease progression or death. Survival analysis will be compared between groups using the log-rank test. Kaplan-Meier estimates of 2-year PFS, DMFS, DSS and OS will be provided for each treatment group and as a post-hoc subgroup analysis based on patient characteristics described above. Median follow-up time will be derived using both complete and incomplete follow-up times. Cox proportional hazards regression will be used to provide hazard ratio estimates. A *p*-value of less than 0.05 will be considered statistically significant. For the evaluation of biomarkers on one time point, differences between groups will be tested using the Mann-Whitney U test. For the evaluation of biomarkers over time, differences between groups will be tested using the Wilcoxon signed-rank test. To evaluate correlations, Spearman correlation coefficients will be calculated. A p-value of less than 0.05 will be considered statistically significant.

All statistical analyses will be performed using SPSS (SPSS Inc., Chicago, Il, USA).

## Discussion

After diagnosis of non-metastatic MIBC, patients undergo primary staging followed by neo-adjuvant chemotherapy (if eligible) and radical local treatment. The prognosis of MIBC remains poor, despite this aggressive treatment [3, 4]. Several reports indicate the superiority of ^18^F-FDG-PET-CT compared to CT in the detection of lymph node and distant metastases [7]. Furthermore, there is evidence that the ^18^F-FDG-PET-CT result is correlated with OS and DSS [8]. We hypothesize that the additional information of ^18^F-FDG-PET-CT can be used to guide local treatment in case of presence of pelvic nodes metastases and that the presence of extra-pelvic metastases on ^18^F-FDG-PET-CT enables MDT or necessitates the early initiation of immunotherapy. The aim of this prospective phase II trial is to evaluate the impact of implementing a ^18^F-FDG-PET-CT in stratifying patients with primary MIBC and tailoring the treatment accordingly in order to improve the patient’s outcome.

## Supplementary Information

**Additional file 1.** Items from the World Health Organization Trial Registration Data Set.

**Additional file 2.** ^18^F-FDG-PET-CT procedure. Description of the ^18^F-FDG-PET-CT procedure.

**Additional file 3.** Stereotactic body radiation therapy (SBRT) procedure, conducted as metastasis directed therapy (MDT) in treatment arm 2.

## Data Availability

The data set used and/or analyzed during the current study are available from the corresponding author on reasonable request. Not all data are obtained yet since the study is still ongoing.

## Abbreviations

^18^F-FDG: 18F-fluorodeoxyglucose
AAPM: The American Association of Physicists in Medicine
AE: Adverse events
CBCT: Cone beam computed tomography
CT: Computed tomography
CTCAE: Common Terminology Criteria for Adverse Events
DMFS: Distant metastasis-free survival
DSS: Disease-specific survival
EORTC: European Organisation for Research and Treatment of Cancer
GTV: Gross tumor volume
Gy: Gray
IMRT: Intensity-modulated radiotherapy
mCi: Millicurie
MDT: Metastasis-directed therapy
MIBC: Muscle-invasive bladder cancer
MRI: Magnetic resonance imaging
OAR: Organs at risk
OS: Overall survival
PET-CT: Positron emission tomography-computed tomography
PFS: Progression-free survival
PRV: Planning risk volume
PTV: Planning target volume
QOL: Quality of life
RC: Radical cystectomy
RECIST: Response Evaluation Criteria in Solid Tumors
SBRT: Stereotactic body radiation therapy
TMT: Trimodality therapy
TURb: Transurethral resection of the bladder
VMAT: Volumetric modulated arc therapy
