# Supplementary material for: Evaluating the impact of 18F-FDG-PET-CT on risk stratification and treatment adaptation for patients with muscle-invasive bladder cancer (EFFORT-MIBC): a phase II prospective trial
Source: BMC Cancer. 2021 Oct 18;21:1113. doi: 10.1186/s12885-021-08861-x (PMC8522089; doi:10.1186/s12885-021-08861-x)
Supplement: Supplementary file 1 — Additional file 1. Items from the World Health Organization Trial Registration Data Set. [file 12885_2021_8861_MOESM1_ESM.docx]

| **Data category** | **Information** |
| --- | --- |
| Primary Registry and Trial identifying Number | ClinicalTrials.gov  NCT04724928 |
| Date of Registration in Primary Registry | Jan 21, 2021 |
| Secondary Identifying Numbers | BC-07456, PK-0061377 |
| Source(s) of Monetary or Material Support | Kom op tegen Kanker (Stand up to Cancer) |
| Primary Sponsor | Ghent University Hospital, Belgium |
| Contact for Public/scientific Queries | *VF* MD, PhD, valerie.fonteyne@uzgent.be, C. Heymanslaan 10, B-9000 Ghent, Belgium |
| Public/scientific Title | Evaluating the impact of 18F-FDG-PET-CT on risk stratification and treatment adaptation for patients with muscle-invasive bladder cancer (EFFORT-MIBC): a phase II prospective trial |
| Countries of Recruitment | Belgium |
| Health Condition(s) or Problem Studied | Muscle-invasive bladder cancer |
| Interventions | - Imaging: 18F-FDG-PET-CT (in addition to and at the timing of the conventional imaging)  -Metastasis directed therapy:   - Surgery: metastasectomy of maximally 3 metastatic lesions - Radiation: Stereotactic body radiotherapy is administered to maximally 3 lesions in 3 fractions of 10Gy   -Immunotherapy: depending on the applicable guidelines |
| Inclusion and exclusion criteria | Inclusion: Histopathology-proven MIBC on TURb or ≥T3 on conventional imaging treated with MIBC radical treatment; T1-4 N0-3 M0 MIBC on conventional imaging (abdominopelvic and thoracic CT/ MRI); Age >18 years; WHO 0-2; Willingness to undergo 18F-FDG-PET-CT; Willingness to undergo MDT or immunotherapy in case of diagnosis of oligometastatic or polymetastatic disease on either 18F-FDG-PET-CT, respectively; Willingness and ability to provide a signed informed consent according to ICH/GCP and national/local regulations  Exclusion: Presence of distant metastasis on conventional imaging (abdominopelvic and thoracic CT/ MRI); Refusal of or having contraindications to 18F-FDG-PET-CT; Refusal of MDT or immunotherapy; Prior radiotherapy unabling MDT; Contraindications to radiotherapy (including active inflammatory bowel disease); Contraindications to immunotherapy; Other primary tumor diagnosed <5 years ago and for which treatment is still required, except for diagnosis of non-metastatic prostate cancer at time of diagnosis of MIBC or non-melanoma skin cancer. |
| Study Type | Interventional, non-randomised, parallel assignment, phase 2 |
| Date of First Enrollment | April 27, 2021 |
| Sample Size | 156 |
| Recruitment Status | Recruiting |
| Study sites | Consult ClincicalTrials.gov for most recent status study sites |
| Primary Outcome(s) | Overall survival |
| Key Secondary Outcomes | progression-free survival, distant metastasis-free survival, disease-specific survival, the added diagnostic value of 18F-FDG-PET-CT compared to conventional imaging |
| Ethics Review | Ethics Committee of the Ghent University Hospital (BC-07456) approved this study on 11/5/2020 |
