# Supplementary material for: Evaluating the impact of 18F-FDG-PET-CT on risk stratification and treatment adaptation for patients with muscle-invasive bladder cancer (EFFORT-MIBC): a phase II prospective trial
Source: BMC Cancer. 2021 Oct 18;21:1113. doi: 10.1186/s12885-021-08861-x (PMC8522089; doi:10.1186/s12885-021-08861-x)
Supplement: Supplementary file 2 — Additional file 2. 18F-FDG-PET-CT procedure. Description of the 18F-FDG-PET-CT procedure. [file 12885_2021_8861_MOESM2_ESM.docx]

^18^F-FDG-PET-CT procedure

Whole-body (skull to mid-thighs) PET images will be acquired just after (dynamic phase) and in 60 min (+/- 15 min) after injection of (patient body weight/10 *1,5) +1 mCi ^18^F-FDG. Blood glucose levels will be measured and should be below 200 mg/dl at injection time. The dynamic acquisition will be performed during the first 10 min after injection at a rate of 2 min/frame. If possible, the patient should be positioned with the arms elevated above the head to avoid beam-hardening artefacts in the abdominal and pelvic region as well as artifacts caused by truncation of the measured field of view. If the ^18^F-FDG-PET-CT data are used for radiation planning the examination should be performed in the same position as used during radiotherapy and the patient will be positioned on a special bed to accomplish this. A minimum of 3 minutes per bed position will be used. Whole-body anatomic imaging through CT-scan will be acquired in the same session. Contrast may be administered if requested by the referring clinician and is decided site dependent. The PET-scan will be corrected using attenuation data of the anatomic imaging.
