# Supplementary material for: Evaluating the impact of 18F-FDG-PET-CT on risk stratification and treatment adaptation for patients with muscle-invasive bladder cancer (EFFORT-MIBC): a phase II prospective trial
Source: BMC Cancer. 2021 Oct 18;21:1113. doi: 10.1186/s12885-021-08861-x (PMC8522089; doi:10.1186/s12885-021-08861-x)
Supplement: Supplementary file 3 — Additional file 3. Stereotactic body radiation therapy (SBRT) procedure, conducted as metastasis directed therapy (MDT) in treatment arm 2. [file 12885_2021_8861_MOESM3_ESM.docx]

Stereotactic body radiation therapy procedure

*Simulation*: Patients receive a planning-CT in supine position with 3 mm CT slice thickness through the tumor site. The planning simulation should cover the target and all organs at risk (OAR). A typical scan length should extend at least 10 cm superior and inferior beyond the treatment field borders. Support devices to increase patient comfort will be chosen depending on the tumor localization. Lung and liver tumor sites will be simulated with 4D-CT, considering breathing.

*Target definition*: The gross tumor volume (GTV) of the suspicious metastases is defined by the physician as all known gross disease visualised on the ^18^F-FDG-PET-CT scan. The planning target volume (PTV) is created using a minimal margin of 3mm in all directions around the GTV.

*Organs at risk definition:* A planning risk volume (PRV) is mandatory for nearby OAR and margins should be as per institutional policy. A PRV expansion of at least 5 mm is suggested for bladder, rectum, sigmoid, colon and intestine, and dose constraints apply to this PRV.

*Dose constraints*: Dose to OAR for SBRT are stipulated in the AAPM report 101 – 3 fraction schedule by *Benedict et al*. It is strongly recommended that dose constraints are not exceeded. If a dose constraint cannot be achieved due to overlap of the target with an OAR or PRV, the dose per fraction can be lowered or the target coverage compromised in order to meet the constraint.

*Treatment technique*: Intensity-modulated radiotherapy (IMRT) or use of rotational techniques is mandatory. Only dosimetry obtained by inversed treatment planning is considered as IMRT. IMRT may be performed by using Step-and-Shoot-Technique, Sliding-Window-Technique or volumetric modulated arc therapy (VMAT). 3D conformal techniques are not allowed in this trial.

*Dose prescription*: A total dose of 30 Gy (80% of the maximal dose) will be delivered in 3 fractions and fractions will be separated >48h and <96h. Treatment will be prescribed to the periphery of the target (80% of the dose (=30Gy), should cover 90% of the PTV). In case of violation of dose constraints to the surrounding OAR, the prescription will be adapted accordingly.

*Radiotherapy delivery and verification*: Treatment will be delivered with static or rotational IMRT with 6-18 MV photons of a linear accelerator using cone beam CT (CBCT) set-up and on-line correction of patient’s position. If multiple targets are irradiated and the targets are more than 10 cm apart in the cranio-caudal direction, multiple isocenters are needed with a CBCT prior to every treatment for every isocenter. Patient immobilization devices can be used according to the institutional policy.

*Duration of therapy*: Patients may discontinue protocol therapy when unacceptable toxicity is encountered. Patients may also discontinue protocol in case of intercurrent illness which would in the judgment of the investigator affect patient safety, the ability to deliver treatment or by request of the patient.
